# Spider Chitin: An Ultrafast Microwave-Assisted Method for Chitin Isolation from *Caribena versicolor* Spider Molt Cuticle

**DOI:** 10.3390/molecules24203736

**Published:** 2019-10-16

**Authors:** Tomasz Machałowski, Marcin Wysokowski, Mikhail V. Tsurkan, Roberta Galli, Christian Schimpf, David Rafaja, Erica Brendler, Christine Viehweger, Sonia Żółtowska-Aksamitowska, Iaroslav Petrenko, Katarzyna Czaczyk, Michael Kraft, Martin Bertau, Nicole Bechmann, Kaomei Guan, Stefan R. Bornstein, Alona Voronkina, Andriy Fursov, Magdalena Bejger, Katarzyna Biniek-Antosiak, Wojciech Rypniewski, Marek Figlerowicz, Oleg Pokrovsky, Teofil Jesionowski, Hermann Ehrlich

**Affiliations:** 1 Institute of Chemical Technology and Engineering, Faculty of Chemical Technology, Poznan University of Technology, 60965 Poznan, Poland; tomasz.g.machalowski@doctorate.put.poznan.pl (T.M.); marcin.wysokowski@put.poznan.pl (M.W.); soniazolaks@gmail.com (S.Ż.-A.); 2 Institute of Electronics and Sensor Materials, TU Bergakademie Freiberg, 09599 Freiberg, Germany; iaroslavpetrenko@gmail.com (I.P.); andriyfur@gmail.com (A.F.); 3 Leibniz Institute of Polymer Research Dresden, 01069 Dresden, Germany; tsurkan@ipfdd.de; 4 Clinical Sensoring and Monitoring, Department of Anesthesiology and Intensive Care Medicine, Faculty of Medicine, TU Dresden, 01307 Dresden, Germany; roberta.galli@tu-dresden.de; 5 Institute of Materials Science, TU Bergakademie Freiberg, 09599 Freiberg, Germany; Christian.Schimpf@iww.tu-freiberg.de (C.S.); David.Rafaja@ww.tu-freiberg.de (D.R.); 6 Institute of Analytical Chemistry, TU Bergakademie Freiberg, 09599 Freiberg, Germany; Erica.Brendler@chemie.tu-freiberg.de (E.B.); Christine.Viehweger@chemie.tu-freiberg.de (C.V.); 7 Department of Biotechnology and Food Microbiology, Poznan University of Life Sciences, 60637 Poznan, Poland; katarzyna.czaczyk@up.poznan.pl; 8 Institute of Chemical Technology, TU Bergakademie Freiberg, 09599 Freiberg, Germany; michael.kraft@chemie.tu-freiberg.de (M.K.); martin.bertau@chemie.tu-freiberg.de (M.B.); 9 Institute of Clinical Chemistry and Laboratory Medicine, University Hospital Carl Gustav Carus, TU Dresden, 01307 Dresden, Germany; Nicole.Bechmann@uniklinikum-dresden.de; 10 Institute of Pharmacology and Toxicology, TU Dresden, 01307 Dresden, Germany; kaomei.guan@tu-dresden.de; 11 Center for Regenerative Therapies Dresden, TU Dresden, 01307 Dresden, Germany; stefan.bornstein@uniklinikum-dresden.de; 12 Department of Medicine III, University Hospital Carl Gustav Carus Dresden, TU Dresden, 01307 Dresden, Germany; 13 Department of Pharmacy, National Pirogov Memorial Medical University, 21018 Vinnytsia, Ukraine; algol2808@gmail.com; 14 Institute of Bioorganic Chemistry, Polish Academy of Sciences, 61704 Poznan, Poland; mbejger@ibch.poznan.pl (M.B.); kbiniek@ibch.poznan.pl (K.B.-A.); wojtekr@ibch.poznan.pl (W.R.); m.figlerowicz@ibch.poznan.pl (M.F.); 15 Geoscience and Environment Toulouse, UMR 5563 CNRS, 31400 Toulouse, France; oleg.pokrovsky@get.omp.eu; 16 BIO-GEO-CLIM Laboratory, Tomsk State University, Lenina St. 36, 634050 Tomsk, Russia

**Keywords:** biopolymers, chitin, microwave, extraction, spider molt cuticle, melanin

## Abstract

Chitin, as a fundamental polysaccharide in invertebrate skeletons, continues to be actively investigated, especially with respect to new sources and the development of effective methods for its extraction. Recent attention has been focused on marine crustaceans and sponges; however, the potential of spiders (order Araneae) as an alternative source of tubular chitin has been overlooked. In this work, we focused our attention on chitin from up to 12 cm-large Theraphosidae spiders, popularly known as tarantulas or bird-eating spiders. These organisms “lose” large quantities of cuticles during their molting cycle. Here, we present for the first time a highly effective method for the isolation of chitin from *Caribena versicolor* spider molt cuticle, as well as its identification and characterization using modern analytical methods. We suggest that the tube-like molt cuticle of this spider can serve as a naturally prefabricated and renewable source of tubular chitin with high potential for application in technology and biomedicine.

## 1. Introduction

The aminopolysaccharide chitin has been recognized as the main structural element in the exoskeletons of a broad diversity of organisms including protists, fungi, diatoms, coralline algae, sponges, corals, annelids, mollusks, and arthropods (for overview see [1,2,3,4,5,6,7,8,9,10]). Recently, the presence of chitin in fish and amphibians has also been debated [11].

**Figure 1:**
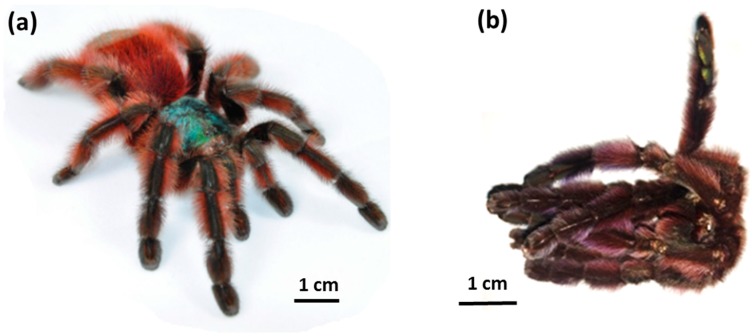
(**a**) Live adult female of the spider *Caribena versicolor* (Theraphosidae) (photo by Krzysztof Siorak), and (**b**) the molt of the same organism as used during this study.

The interest of the scientific community in this structural polysaccharide has bifurcated in two directions. The first concerns the evolution of chitin systems in diverse phyla [8,12,13]. The second is directed towards the biomedical and technological potential of chitin and its derivatives (e.g., chitosan) [14,15,16]. Traditionally chitin has been isolated on a large scale only from fungal biomass (e.g., from the mycelia of *Aspergillus niger*, *Mucor rouxii*, *Agaricus bisporus*) [17,18,19,20,21] and exoskeletons of crustaceans, in the form of powders, whiskers, and flakes [22]. These forms remain excellent sources for the further production of chitosan. However, diverse additional and mostly expensive methods and approaches have been developed purposefully during the last decade to produce open-pore chitin matrices, foams and scaffolds [23], for example, for biomedical applications. Consequently, recent trends in relation to the applicability of chitin for such purposes are based on monitoring of those chitin sources where naturally prefabricated constructs with tube-like and 3D architectures already exist. Most efforts in this case have used microtubular, chitinous 3D scaffolds of marine sponge origin [3,4,9,24,25,26,27]. These constructs have recently been successfully used as adsorbents [28], membranes [29], templates in extreme biomimetics [30,31,32,33,34], as well as biocompatible scaffolds for tissue engineering of chondrocytes [3,35] and human mesenchymal stromal cells [36,37]. Due to the possibility of cultivating marine sponges in marine farming facilities, these invertebrates represent a unique source of renewable, ready-to-use chitinous scaffolds [38,39]. In looking for new sources of naturally prefabricated chitin-based materials, we focused our attention on spiders, which “lose” large quantities of chitin-containing cuticles during their molting cycle (ecdysis) (Figure 1). The main purpose of ‘’changing skin’’ is to enable the growth (10–16%) [40] and further development of the body. Recently, Machałowski et al. [41] evaluated biomedical application of tubular chitin scaffold isolated from *Caribena versicolor* molt for both, human progenitor (hPheo1) and human cardiomyocytes (iPSC-CMs) cells. Moreover, authors used it for the first time as a porous membrane for fabrication of CuO/Cu(OH)_2_/chitin catalyst for the reduction of 4-nitrophenol (4-NP) to 4-aminophenol (4-AM) [41].

Due especially to their extremely interesting coloration and gentle temperament, Theraphosidae spiders are very popular showpieces among hobbyists [42]. The number of such spiders in Europe alone is approximately 2 million (personal communication from INTIB GmbH, Freiberg, Germany). This number was estimated based on official data on legal spider breeders [43,44,45]. To date, 48,053 species of spiders have been reported worldwide [46]. To grow, spiders—like other arthropods—must form a new, larger exoskeleton and shed the old one in a process of ecdysis [40,47,48]. The global spider community, estimated based on average spider biomass per square meter in various terrestrial biomes, amounts to 25 million metric tons fresh weight [49]. Another study indicates that because of ecdysis a spider loses about 8 ± 0.16% of body fresh weight, and this data is constant for all spider species [47]. Based on these figures we may calculate that spiders could produce due to molting between 2 and 6 million tons of cuticle (depending on the frequency of molting) per year worldwide. Surprisingly, despite their diversity and abundance, spiders have received only limited attention with regard to the utilization of their molts as a potential renewable source of chitin. There are still no data confirming the presence of chitin in the molts of Theraphosidae spiders.

All arthropods, including spiders (Araneae), have a body covered with strong exoskeleton [50,51]. In the Araneae order this consists mainly of chitin combined with protein [52,53] (endocuticle) and a non-chitin outermost subdivision (epicuticle) [54]. Chitin crosslinked with protein makes up about 90% [54] of the total organic content of the cuticle. Lipids (wax), polyphenols, enzymes, as well as pigments are other organic constituents which contribute to protecting the spider from dehydration and reduce water loss [54]. Additionally, histochemical and spectroscopic investigations into the silk ducts of the *Nephila edulis* spider indicate that they contain β-chitin. The presence of β-chitin suggests that arthropod silk ducts and glands may have evolved from the trichogen and tormogen of a hollow cuticular spine capable of secreting a non-Newtonian fluid derived from a cuticular protein [55]. Additionally, it is suggested by Davies et al. (2013) [55] that the arrangement of chitin and heavily stabilized matrix prevents unwanted deformations of this region of the duct, whose geometry is thought to be vital for spinning.

Compared with other chelicerates, spiders present a wide range of coloration [54]. New light was shed on spider pigmentation by the discovery of melanin [56]. We also suggest studying the molts of *C. versicolor* as a potential source of melanin-like pigments. Melanin compounds present antioxidant, antitumor [57], antifungal [58], antibacterial [59,60], and anti-inflammatory activity [61]. They have biomedical applications [62] as well as other uses in pharmacy and medicine [63].

**Figure 2:**
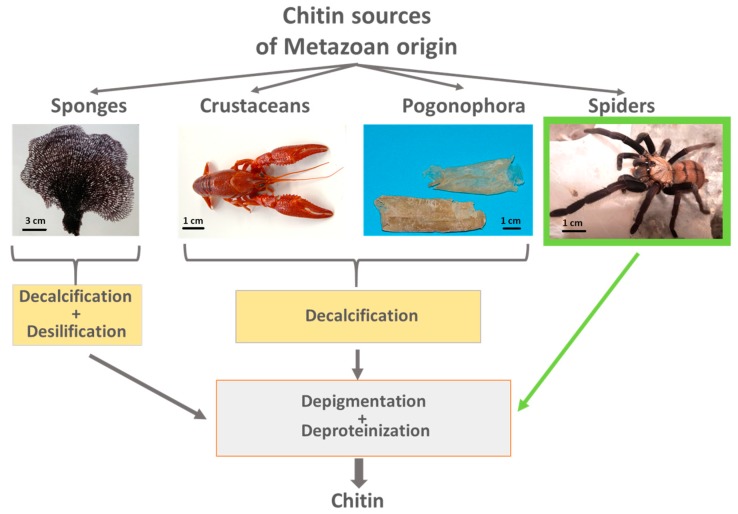
Principles of chitin isolation from diverse sources of metazoan origin.

In contrast to sponges, worms, mollusks and crustaceans (Figure 2), the cuticle of spiders is free from mineral phases like calcium carbonates, with the exception of Zn-based biominerals localized within the fang of some species (see for overview [64,65]). Consequently, in the case of molt cuticles of arachnids, we can omit the demineralization step and begin immediately with the deproteinization and depigmentation procedures.

In this study, we investigated 25 selected molts (ecdysis cuticles) from the (Aviculariinae) tarantula *Caribena versicolor* (Walckenaer, 1837), previously known also as *Avicularia versicolor* [66] (Figure 1b). The natural habitats of this predatory Theraphosidae tarantula are mesophyll forests of Martinique, Guadeloupe, and Dominica [67]. It builds its web nests in Bromeliacea leaves, among tree branches, in abandoned hollows, and next to human constructions. Their life cycle usually lasts about two years for males and about five for females. The species requires around ten molts to reach sexual maturity (approximately 18 months of life) [66].

Thus, the aim of the first part of this study was to develop a highly effective method for isolating chitin and pigment from *C. versicolor* spider molt cuticle, and to perform their identification and characterization using well-recognized analytical tools such as FTIR and Raman spectroscopy, ^13^C solid state NMR spectroscopy, XRD, ESI-MS, and specific surface area (BET) measurements. We suggest that the residual tube-like molt cuticle which originally covered the legs of this spider (Figure S1, Supplementary Materials) can serve as a naturally prefabricated source for the isolation of tubular chitin, which resembles it in shape, size and morphology, including porosity. The second part of our study is dedicated to practical applications of the chitin isolated from *C. versicolor* molt cuticles.

## 2. Results and Discussion

There are several publications concerning the structure [68,69], ultrastructure [52,53,70,71,72], and chemistry [73,74,75,76,77] of cuticles of diverse arachnid origin. Despite the existence of a few differences, all spiders possess the following common features: (a) the outermost layer of cuticle (epicuticle) contains lipoproteins and no chitin; (b) the procuticle, however, is based on a chitin–protein complex. The cuticle of spiders’ walking legs represents a complex system of pore and wax canals that connect the epidermis with the cuticle surface [74]. It is well recognized that chloroform: ethanol treatment does not significantly alter the morphology of any of the cuticular layers or the contents of the wax/pore canals [74].

**Figure 3:**
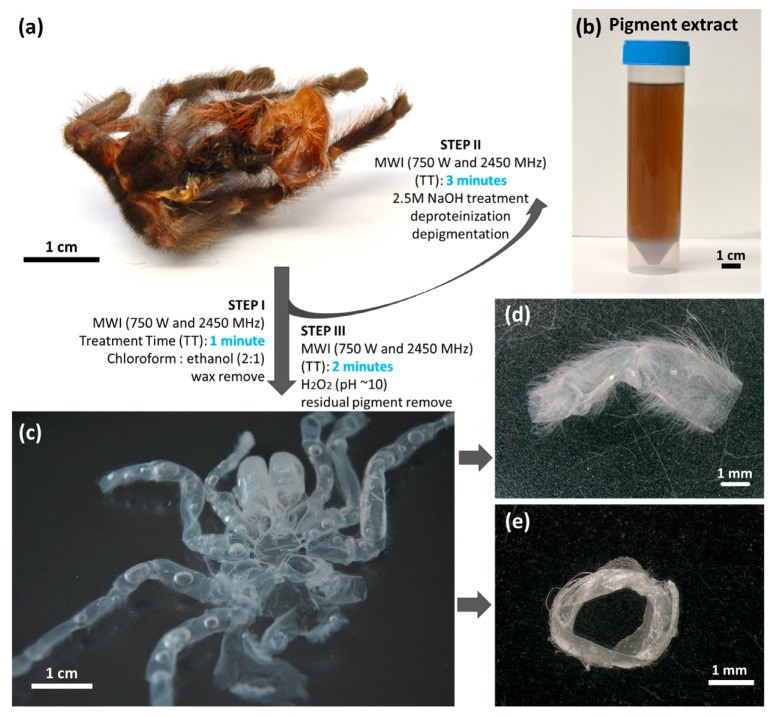
Schematic view of the microwave-assisted method (MWI) for both chitin and pigment isolation from (**a**) the molt cuticle of the spider *C. versicolor*. (**b**) Brownish colored pigment extract was obtained after alkali-based treatment. (**c**) The pigment-free, translucent molt represents a source of prefabricated tubular chitin that resembles the shape, (**d**,**e**) size, and morphology of the spider’s walking legs. See also Figure 4.

**Figure 4:**
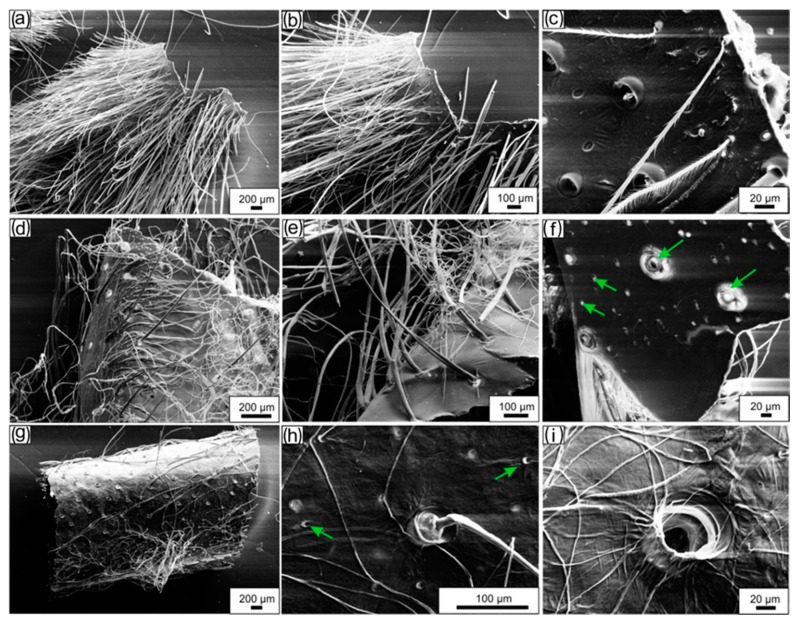
SEM imagery. (**a**,**b**) A fragment of the naturally occurring molt cuticle obtained from the walking leg of *C. versicolor* spider represents a brush-like tube. (**c**) The specific structure of individual setae as well as pores on the surface of the molt are well visible on the image. (**d**,**e**) Lipid-, wax-, and pigment-free cuticle resembles the size, shape, and morphology of the molt. (**f**) Pores (arrows) are well visible also on the inner surface of the tube-like construct. Microwave-assisted treatment of the molt as represented in (**a**) leads to partial removal of the setae (**g**). (**h**,**i**) Pores of diverse diameter (arrows) remain visible.

After the isolation process was completed, transparent exoskeleton from *C. versicolor* was obtained (Figure 3c). Based on a calculation (Equation (1)) the content of chitin in *C. versicolor* was determined at 19% of the molt by mass. The morphological features of *C. versicolor* walking leg cuticle before and after treatment according to the procedures represented in Figure 3 are well visible in the SEM images (Figure 4).

**Figure 5:**
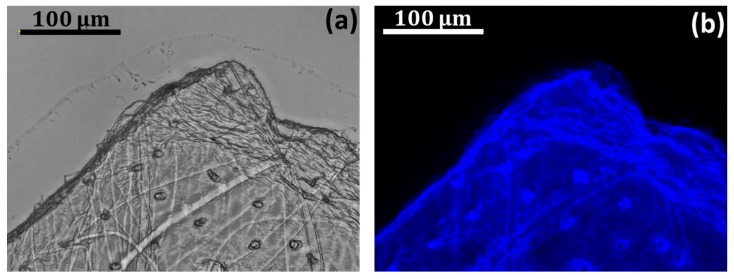
(**a**) Light microscopy and (**b**) fluorescence microscopy images of a selected fragment of the porous cuticle isolated from *C. versicolor* and stained with CFW. (**b**) Light exposure time in image: 1/6800 s.

We began the identification of chitin in selected fragments of purified cuticle using Calcofluor White staining (CFW). This method is well recognized for the identification of polysaccharides based on β-glycosidic bonds, including chitin. The intensive blue fluorescence is measurable even under a light exposure time of 1/6800 s (Figure 5). This result is typical for chitin staining, as reported previously [3,4,78].

The chitinase digestion test using Yatalase® is a highly specific method to confirm the presence of chitin in a material [79,80]. The enzyme is responsible for endo-hydrolysis of *N*-acetyl-β-d–glucosamine-β-(1→4) linkages. The effect of chitinase activity over 6 h against a selected fragment of *C. versicolor* cuticle is represented in Figure S2. Similar results using Yatalase have been reported previously with respect to chitin of poriferan origin [24,26,81,82]. In contrast to Yatalase from Corynebacterium sp. OZ-21, two other chitinases isolated from *Moritella marina* and *Pyrococcus chitonophagus* were inactive against spider chitin (see Section 3.3.9 and Supplementary Materials).

**Figure 6:**
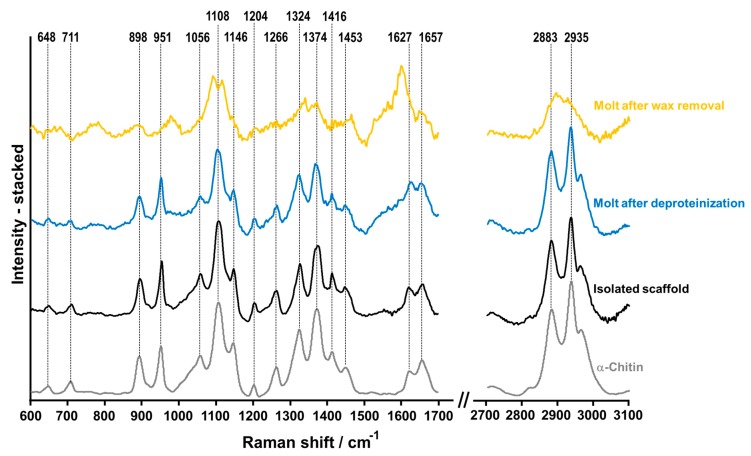
Raman spectra of cuticle after lipid removal (yellow line), molt cuticle after deproteinization (blue line), chitin scaffold from *C. versicolor* spider isolated using the microwave-assisted approach (black line) and α-chitin standard (gray line).

Both Raman spectroscopy (Figure 6) and ATR-FTIR (results described in Supplementary Materials, Figure S3) spectroscopy are currently considered highly sensitive and useful techniques for the identification of carbohydrates (especially polysaccharides) [83]. It is worthwhile to note that vibrational spectroscopy also gives detailed insights into intermolecular interactions between natural polysaccharides and proteins [84]. Comparative analysis of the results obtained with Raman spectroscopy (Figure 6) shows that the spectrum recorded for the isolated chitinous scaffold of *C. versicolor* corresponds to the spectrum acquired for the α-chitin standard. Aliphatic stretching features are visible at 2935 cm^−1^ and 2883 cm^−1^, and the corresponding bending modes of these groups are registered at 1453 cm^−1^ and 1374 cm^−1^ [85]. The existence of two peaks characteristic for the amide I band at *v*_max_ 1657 and 1627 cm^−1^, as well as an intense band related to the β-glycosidic bond at *v*_max_ 898 cm^−1^, clearly indicate that the isolated chitin consists of the α isomorph [24,86]. Peaks at 1324 cm^−1^ and 1266 cm^−1^ are associated with the C–N stretching and N–H deformation of the amide III band. The band recorded in the range 950–1108 cm^−1^ corresponds to C–O and C–C vibrations of the saccharide ring [6].

**Figure 7:**
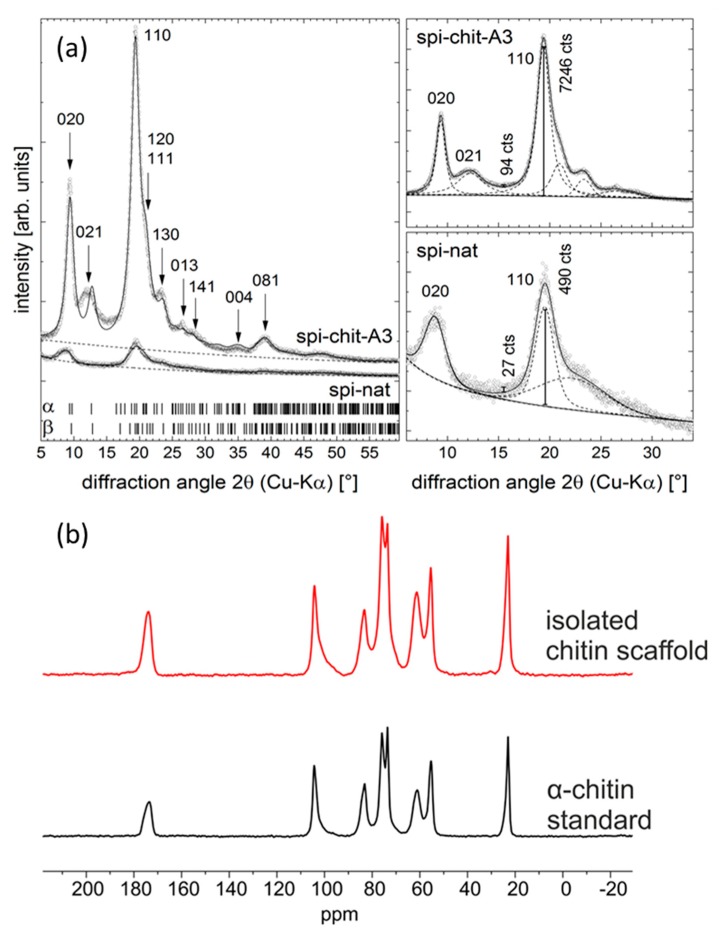
(**a**) Diffraction pattern of samples of natural molt cuticle (spi-nat) and isolated chitin scaffold (spi-chit-A3). The good match between experimental (dots) and calculated data (line) provides evidence for the presence of α-chitin (left, Rietveld refinement, phase identification). For comparison, the theoretical line positions for α- and β-chitin are also shown. The Laue indices of the most prominent diffraction maxima are given. The refinement involved a preferred orientation of α-chitin. The intensity measures used for the determination of the CrI are indicated in the patterns shown on the right (single peak fitting, dashed lines = individual peaks, solid line = sum profile, vertical bars = I_am_ and I_110_ determined at 2θ ≈ 16° and 2θ ≈ 19.5° respectively). (**b**) ^13^C CP/MAS NMR spectra of isolated chitin scaffold (red line) and α-chitin (black line).

X-ray diffraction (XRD) was used to probe the structure of the extracted chitin. Generally, chitin may occur in two modifications, α [87] and β [88]. Sometimes a third, γ, is also considered, but it may be regarded as a mixture of α and β [89]. α-chitin crystallizes in the orthorhombic space group P2_1_2_1_2_1_ with lattice parameters a = 4.750 Å, b = 18.890 Å, c = 10.333 Å [90]. β-chitin crystallizes in the less symmetric space group P2(1) with a = 4.819 Å, b = 9.239 Å, c = 10.384 Å, β= 97.16° [88]. For the identification of the chitin type, the CIF files provided in [87] and [88] were used. The diffraction patterns in Figure 7a are taken from studied samples. The measured data are indicated as small dots, while the refinement is shown as a solid line. The refinement shown in the left part of Figure 7a was a Rietveld-like pattern refinement using TOPAS software (Bruker AXS, Karlsruhe, Germany), having the purpose of phase identification. The diffraction patterns of α- and β-chitin are very similar with respect to the diffraction line positions (cf. theoretical line positions as indicated in Figure 7a left) but can be distinguished based on the relative peak intensities. The latter makes a full powder pattern fitting nearly inevitable for phase identification.

A special feature of the refinement was the inclusion of a preferred orientation of the spherical harmonic type (6^th^ order) as implemented in TOPAS. This preferred orientation may result from the fiber-like texture of the chitin and from the sample preparation. However, strong indication of the presence of α-chitin is provided by the small diffraction maxima visible in the angular range 2θ = 20–25°, which are absent for β- and γ-chitin. Furthermore, all refinement trials gave better ‘goodness-of-fit’ values for α-chitin than for β-chitin. In Figure 7a, the Laue indices of the most prominent diffraction lines corresponding to α-chitin are given. This analysis leads to the conclusion that the majority of the chitin produced here is α-chitin. Given the large line widths of the peaks and the similarities in the diffraction patterns of α- and β-chitin, small amounts of β-chitin may be present, but cannot be reliably identified. The right part of Figure 7a represents individual peak fits, serving to check the amorphous-to-crystalline ratio of the samples. Again, TOPAS software was used to obtain the profiles of the individual peaks.

The crystalline index (CrI; %) was calculated according to Equation (5) (see Section 3.3.6) I_110_ is the peak height of the 110 reflection at 2θ ≅ 19.2° and I_am_ is the intensity of an amorphous ‘hump’ underlying the diffraction pattern, approximately from the 020 to the 060 reflection (d ≈ 10, 3 Å, for Cu-radiation: 2θ ≈ 8, 30°). This latter ‘maximum’ was not fitted but was estimated from the ‘missing intensity’ of the calculated pattern. In this way, we estimated I_am_ at 2θ ≅ 15.5°. Although the approximation of the crystalline fraction of the fibers is rather crude [91], its applicability has been demonstrated for cellulose [92], chitosan [93], and chitin [94,95], amongst others. Using the intensity values given in Figure 7a (right) at the positions indicated by the vertical bars, the CrI values for the samples were calculated by Equation (5) using the intensity counts shown in the figures: spi-nat: 94%, spi-chit-A3: 99%. This confirms that the proposed MWI does not have a negative influence on the crystallinity of chitin.

^13^C solid state NMR spectroscopy is also a useful technique to obtain structural information about the isolated chitin scaffold and enables an effective comparison with α-chitin. The results are given in Figure 7b. The ^13^C CP/MAS NMR spectrum of the investigated α-chitin (black line) shows eight ^13^C signals in the range 0 ppm to 200 ppm, which corresponds to known chemical shift data for α-chitin [96]. The spectrum of the isolated chitin scaffold (red line) is similar to that of α-chitin, which permits the conclusion that there is structural conformity between the isolated chitin scaffold and α-chitin. Likewise, both samples show similar structural homogeneity, due to well-resolved resonances in both ^13^C CP/MAS NMR spectra. There are no signs of the existence of other structural elements in the ^13^C CP/MAS NMR spectrum of the isolated chitin scaffold.

BET measurements (Figure S4, Supplementary Materials) revealed that the natural cuticle had a specific surface area greater by a factor of two than that of the spider chitin (17.0 ± 1.5 and 9.00 ± 0.78 m^2^/g, respectively).

**Figure 8:**
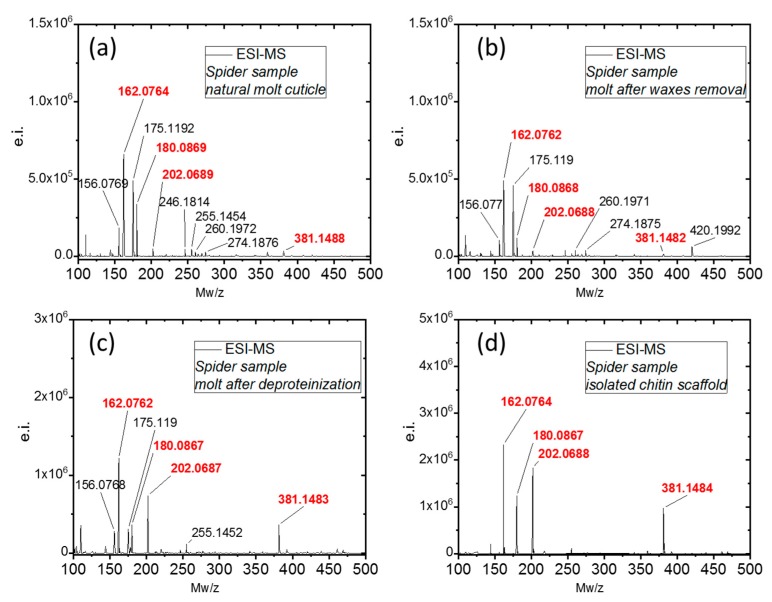
ESI-MS investigations of the chitin isolated from *C. versicolor* spider: (**a**) natural molt cuticle; (**b**) molt after wax removal; (**c**) molt after deproteinization; (**d**) isolated chitin scaffold.

Acetic hydrolysis of chitin by strong acids resulted in the formation of D-glucosamine (dGlcN), which can be observed in ESI-MS spectroscopy. This method is a standard for chitin identification and has been utilized for chitin discovery in various organisms [39,97] as well as fossil remains [98]. Moreover, the quality of the spectrum in terms of signal composition and intensity can provide quick information about the purity of a sample [26]. ESI-MS of d-glucosamine from natural sources typically shows four main signals with *m*/*z* = 162.08, 180.08, 202.07, and 381.14, which correspond to the [M − H_2_O + H^+^], [M + H^+^], [M + Na^+^], and [M − H_2_O + H^+^] species, respectively. The presence of any additional molecular signals is indicative of impurities. As can be seen in Figure 8a,b the samples of natural molt cuticle and the molt after wax removal show the presence of many additional signals, most likely indicating the significant presence of proteins. The removal of the protein content by deproteinization resulted in a ‘cleaner’ spectrum of dGlcN (Figure 8c). The use of the described protocol for chitin scaffold isolation resulted in a spectrum (Figure 8d) which indicates a very high purity of dGlcN.

## 3. Materials and Methods

### 3.1. Materials

Molting cuticles of spiders of *Caribena versicolor* (Figure 1b and Figure S1) were collected from the Wojciech Pałasz tarantula breeding facility, Poland. No permits were required for the described research, which complied with all relevant regulations. The species used in these experiments is not an endangered or protected species under CITES regulations.

### 3.2. Chitin Extraction

#### 3.2.1. Microwave-Assisted Chitin Isolation

The molt of *C. versicolor* was used for microwave-assisted chitin isolation. The procedure of chitin extraction is shown schematically in Figure 3. In the first step, the lipids and waxes were removed from the cuticle surface by treatment with a chloroform:ethanol (*v*/*v* 2:1) mixture under microwave irradiation (MWI) for 1 min (750 W and 2450 MHz). Afterwards, the cuticle was carefully isolated and washed with absolute ethanol. Subsequently, in the second step the sample was treated with 2.5 M NaOH solution and irradiated with microwaves for 3 min. During this step, the temperature of the solution increased to 95 °C, and the solution become orange. The insoluble residue was carefully separated from the solution, washed with distilled water to neutral pH and used in the next step. The last stage was depigmentation performed under MWI for 2 min using 30% H_2_O_2_ (pH~10; pH was regulated by addition of 2.5 M NaOH). Finally, the isolated transparent and soft scaffold (Figure 3c–e) was washed with distilled water to neutral pH and placed in a refrigerator for storage (4 °C).

#### 3.2.2. Estimation of Chitin Content in Molt

For the determination of chitin content in the spider molt, the mass of dry exoskeleton was determined before isolation. After pigment and protein extraction the mass of the dried chitinous molt was determined by weighing. Chitin content was determined based on the differences between these two data, and was calculated using the following formula (Equation (1)):

Chitin content (%) = (m_ch_/m_m_) × 100% (1)

where: m_ch_—the mass of the dry spider chitin, g; m_m_—the mass of dry molt, g.

#### 3.2.3. Pigment Isolation

The extract of pigments obtained after alkali treatment (pH~14) of spider cuticle (Figure 3, step II) using the microwave-assisted method (Section 3.2.1) was first subjected to dialysis (MWCO = 14,000 DA, Roth, Germany). The process was carried out for 12 h and led to pH~7. To remove insoluble residuals, the pigment extract was centrifuged using a Fresco 21 Centrifuge (Thermo Fisher Scientific, Waltham, MA, USA) at 15,000 rpm for 10 min. Finally, the extract solution was dried at 37 °C for 24 h, and the pigment was then collected in the form of powder and used for analytical investigations. For determination of the pigment content in the spider molt, the mass of the dry exoskeleton was determined before and after pigment extraction, and the following formula was applied (Equation (2)):

Pigment content (%) = (m_p_/m_m_) × 100% (2)

where: m_p_—the mass of the obtained pigment powder, g; m_m_—the mass of dry molt, g.

### 3.3. Characterization of Obtained Materials

#### 3.3.1. Light, Fluorescence, and Stereo Microscopy

The chitin exoskeleton isolated from *C. versicolor* was observed using a BZ-9000 (Keyence, Osaka, Japan) microscope in the fluorescent and light microscopy modes. Photographic images were taken with a Keyence VHX-5000 digital optical microscope (Keyence, Osaka, Japan) and a VH-ZST swing-head zoom lens (magnification up to 2000×).

#### 3.3.2. Calcofluor White Staining

The chitin isolated from *C. versicolor* molt was stained with calcofluor white (CFW) (Fluorescent Brightener M2R, Sigma-Aldrich, St. Louis, MO, USA) and compared with an unstained sample. For the staining process, 30 µL of a solution of 10 g glycerin and 10 g NaOH in 90 mL water was applied. After 15 s, the CFW was added, and the material was kept for 6 h without light at 25 °C. It was then washed with distilled water to eliminate the unattached calcofluor white, and dried at 25 °C. On binding to polysaccharides containing β-glycosidic bonds (such as chitin), this fluorochrome secretes a bright blue light under UV excitation even with a very short light exposure time [38].

#### 3.3.3. Scanning Electron Microscopy (SEM)

The microstructure of the materials used in this study was analyzed with the use of a Hitachi S4700 scanning electron microscope (Hitachi Scientific Ltd., Tokyo, Japan). Before analysis the samples were freeze-dried and were then covered with carbon, using an Edward Sputter Coater S150B.

#### 3.3.4. Attenuated Total Reflectance Fourier Transform Infrared Spectroscopy

Infrared spectroscopy was used for the qualitative characterization of the isolated materials. The samples were analyzed using a Nicolet 210c spectrometer (Thermo Fisher Scientific, Waltham, MA, USA). The degree of acetylation (DA) was calculated using the following formula (Equation (3)) [99]

DA% = [(A_1654_/A_3432_)∙100%)]/1.33 (3)

The degree of deacetylation (DD) was calculated according to the following formula (Equation (4)) [100]:

DD% = 100% − DA%(4)

#### 3.3.5. Raman Spectroscopy

Raman spectroscopy was performed using a Raman spectrometer (RamanRxn1™, Kaiser Optical Systems Inc., Ann Arbor, MI, USA) coupled to a light microscope (DM2500 P, Leica Microsystems GmbH, Wetzlar, Germany). A diode laser emitting at a wavelength of 785 nm was used for excitation of the Raman scattering. The laser beam was propagated to the microscope with a 100 µm optical fiber and focused on the samples by means of a 20 ×/0.45 microscope objective, leading to a focal spot of about 50 µm. The Raman signal was collected in reflection configuration and sent to the f/1.8 holographic imaging spectrograph using a 62.5 µm core optical fiber. The spectral resolution, in the range 150–3250 cm^–1^, was 4 cm^–1^. Accumulations of 2 s were used. Depending on the signal quality, 4 to 40 accumulations were added to increase the s/n ratio.

#### 3.3.6. X-ray Diffraction (XRD)

The phase compositions of selected samples were determined using X-ray diffraction. The XRD patterns were recorded using a Seifert-FPM URD6 diffractometer (Seifert-Freiberger Präzisionsmechanik, Freiberg, Germany). The source of X-rays was a sealed X-ray tube with Cu anode that was operated at 30 mA and 40 kV. The fluorescence radiation and other parasitic radiation components were eliminated by a graphite monochromator located in the diffracted beam. All measurements were conducted in Bragg–Brentano geometry. The diffraction signal was detected by a scintillation counter. The samples were fixed with Vaseline onto zero-background holder ((510)-oriented Si single crystal). The presence of identified phases was verified by comparing the measured XRD patterns with XRD patterns calculated for the respective phase mixture. Peak positions were taken from the ICDD-PDF database (PDF 4+) and pattern refinements were performed with the Bruker Topas 5 software (Bruker AXS). The crystalline index (CrI; %) was calculated according to Equation (5) [95]:

CrI_110_ = [(I_110_ − I_am_)/I_110_]∙100% (5)

where: I_110_—the maximum intensity at 2θ ≅ 19.2°; I_am_—the intensity of amorphous diffraction at 2θ ≅ 16°.

#### 3.3.7. Electrospray Ionization Mass Spectrometry (ESI-MS)

The samples were hydrolyzed in 6M HCl for 24 h at 90 °C, and the obtained solution underwent filtration using a 0.4 micron filter to remove solid residues (if present). Next, the solutions were freeze-dried to remove excess HCl. The solid residues were dissolved in water for ESI-MS analysis. The standard d-glucosamine control was purchased from Sigma-Aldrich (St. Louis, MO, USA). All ESI-MS measurements were performed on an Agilent Technologies 6230 TOF LC/MS spectrometer (Applied Biosystems, Santa Clara, CA, USA). Nitrogen was used as the nebulizing and desolvation gas. Graphs were generated using Origin 8.5 for PC (Originlab Corporation, Northampton, MA, USA).

#### 3.3.8. ^13^C Solid State NMR

^13^C cross polarization (CP) spectra were recorded on a Bruker Avance HD 400 MHz WB spectrometer using a 4 mm triple resonance CP MAS probe (1H, 400.30 MHz; ^13^C, 100.67 MHz). A spinning rate of 10 kHz was used. CP experiments were carried out with 1 ms contact time and a 70% ramp. During acquisition time tppm15 decoupling was applied. The recycle delay was set to 5 s. In each case, 1024 scans were taken. The chemical shifts are reported relative to tetramethylsilane (TMS).

#### 3.3.9. Chitinase Digestion Test

Selected fragments of isolated tubular chitin from *C. versicolor* were treated with Yatalase enzyme solution (pH 6.5). The treatment was carried out for 6 h at 37 °C, and the progress of digestion was monitored under light microscopy using a BZ-9000 microscope (Keyence, Osaka, Japan).

Multiple samples of spider exoskeleton, including fragments of cuticle and setae, were incubated with four chitinases: Chi60 from *M. marina* and its deletion mutant, missing two Ig-like domains [101] and two chitinases, Chiton_1119 and Chiton_1716, from *P. chitonophagus* [102]. The samples were incubated with the enzymes at 18, 28, and subsequently 35 °C, for at least 48 h at each temperature. The pH was adjusted to the optimum for the activity of each enzyme: pH 5.0 for Chi60 and its mutant, pH 6.5 for the thermophilic enzymes. Each sample was supplemented with 50 μL of methoxyamine (20 mg/mL in dry pyridine) and vortex-mixed in a thermomixer for 1.5 h at 37 °C; afterwards, it was centrifuged for 10 s. Following the centrifugation the samples were supplemented with 80 μL MSTFA, again vortex-mixed in a thermomixer (30 min, 37 °C) and centrifuged at 11,000× *g* for 10 min. Prepared samples were transferred to inserts at 200 μL. Carbohydrates were analyzed by gas chromatography mass spectrometry (GC-MS, TRACE 1310 GC oven with TSQ8000 triplequad MS from Thermo Fisher Scientific, Waltham, MA, USA) using a DB-5MS column (30 m × 0.25 mm × 0.25 µm, J&W Scientific, Agilent Technologies, Palo Alto, CA, USA). For separation of volatile compounds, temperature gradient was used as follows: 70 °C for 2 min, followed by 10 °C/min up to 300 °C (10 min). For sample injection, PTV injector was used in range of 60 to 250 °C, transfer line temp. was set to 250 °C, source to 250 °C. Spectra were recorded in *m*/*z* range of 50–850 in EI+ mode with electron energy of 70 eV.

#### 3.3.10. UV-VIS Spectroscopy

0.4 mg of pigments isolated from *C. versicolor* cuticle (Figure 3b) was dissolved in 1 mL of 0.1 M KOH, with the assistance of ultrasound treatment, at 80 °C for 30 min. Spectra were obtained for *C. versicolor* pigment and *Sepia officinalis* melanin (Sigma). The spectra were measured with a JASCO V-750 spectrometer in the wavelength range of 200 to 800 nm using a quartz cuvette with path length of 1 cm (quartz suprasil, Hellma Analytics, Müllheim, Germany) and operated at a resolution of 5 nm.

#### 3.3.11. Parameters of the Porous Structure

The BET (Brunauer–Emmett–Teller) specific surface areas (SSAB.E.T) of natural coticule and spider chitin were measured by N_2_ multipoint adsorption technique using the Quantachrome Autosorb Automated Gas Sorption System (Quantochrome Inc., Boynton Beach, FL, USA) with 22 adsorption points (10 to 12 points in the B.E.T. domain) after 163 h of degassing at 120 °C. The typical uncertainty of these measurements was 10%.

## 4. Conclusions

The molt cuticles of bird-eating spiders (Theraphosidae) are highly optimized structures that support and organize the functional tissues and provide important design information for the fabrication of synthetic tubular and porous scaffolds. They represent potential inspirational sources for technology, biomimetics, and biomedicine. Perspectives of applications of *C. versicolor* tubular chitin for the development of a tubular scaffold-based catalyst, as well in tissue engineering of human cardiomyocytes have been mentioned above. In this study, we have presented strong evidence that α-chitin is the main biological material localized within such cuticles and can be isolated from these exoskeletal constructs very effectively in a short time. The rapid isolation of tube-like chitin from Theraphosidae spiders using microwave irradiation with subsequent alkaline and hydrogen peroxide treatments is made possible by the absence of a mineral phase within the naturally occurring molt cuticles. The processing of microgranular chitin—which is obtained industrially from crustaceans—into the desired tube-like materials is technologically difficult as well as expensive because it requires derivatization of chitin to chitosan with use of highly concentrated (40–50%) NaOH and temperature higher than 60 °C. In contrast, chitin-based cuticles of spiders provide almost “ready-to-use” tubular and porous scaffolds for potential applications in tissue engineering and regenerative medicine. These species-specific scaffolds closely resemble the size, shape, and morphology of the original theraphosid spiders. Moreover, these organisms can be recognized as a source of unique, naturally prefabricated chitin even on a large scale, due to their ability to grow under cultivation conditions worldwide. A melanin-related pigment has also been isolated from the spider’s molt cuticles in this study. This compound requires detailed future study with regard to its beneficial properties, including possible antioxidative and antibacterial activity.

## Supplementary Materials

The following information are available: Figures S1–S6.

## References

[1] Bo M., Bavestrello G., Kurek D., Paasch S., Brunner E., Born R., Galli R., Stelling A.L., Sivkov V.N., Petrova O.V. (2012). Isolation and identification of chitin in the black coral *Parantipathes larix* (Anthozoa: Cnidaria). Int. J. Biol. Macromol..

[2] Brunner E., Richthammer P., Ehrlich H., Paasch S., Simon P., Ueberlein S., Van Pée K.H. (2009). Chitin-based organic networks: An integral part of cell wall biosilica in the diatom thalassiosira pseudonana. Angew. Chemie Int. Ed..

[3] Ehrlich H., Steck E., Ilan M., Maldonado M., Muricy G., Bavestrello G., Kljajic Z., Carballo J.L., Schiaparelli S., Ereskovsky A. (2010). Three-dimensional chitin-based scaffolds from Verongida sponges (Demospongiae: Porifera). Part II: Biomimetic potential and applications. Int. J. Biol. Macromol..

[4] Ehrlich H., Malando M., Spindler K.D., Eckert C., Hanke T., Born R., Goebel C., Simon P., Heinemann S., Worch H. (2007). First evidence of chitin as a component of the skeletal fibers of marine sponges. Part I. Verongidae (demospongia: Porifera). J. Exp. Zool..

[5] Erko M., Hartmann M.A., Zlotnikov I., Valverde Serrano C., Fratzl P., Politi Y. (2013). Structural and mechanical properties of the arthropod cuticle: Comparison between the fang of the spider *Cupiennius salei* and the carapace of American lobster *Homarus americanus*. J. Struct. Biol..

[6] Kaya M., Mujtaba M., Ehrlich H., Salaberria A.M., Baran T., Amemiya C.T., Galli R., Akyuz L., Sargin I., Labidi J. (2017). On chemistry of γ-chitin. Carbohydr. Polym..

[7] Rahman H.A., Halfar J. (2014). First evidence of chitin in calcified coralline algae: New insights into the calcification process of *Clathromorphum compactum*. Sci. Rep..

[8] Rudall K.M., Kenchington W. (1973). The chitin system. Biol. Rev..

[9] Wysokowski M., Bazhenov V.V., Tsurkan M.V., Galli R., Stelling A.L., Stöcker H., Kaiser S., Niederschlag E., Gärtner G., Behm T. (2013). Isolation and identification of chitin in three-dimensional skeleton of *Aplysina fistularis* marine sponge. Int. J. Biol. Macromol..

[10] Wysokowski M., Zatoń M., Bazhenov V.V., Behm T., Ehrlich A., Stelling A.L., Hog M., Ehrlich H. (2014). Identification of chitin in 200-million-year-old gastropod egg capsules. Paleobiology.

[11] Tang W.J., Fernandez J.G., Sohn J.J., Amemiya C.T., Tang W.J., Fernandez J.G., Sohn J.J., Amemiya C.T. (2015). Chitin is endogenously produced in vertebrates. Curr. Biol..

[12] Merzendorfer H., Zimoch L. (2003). Chitin metabolism in insects: Structure, function and regulation of chitin synthases and chitinases. J. Exp. Biol..

[13] Zhu K.Y., Merzendorfer H., Zhang W., Zhang J., Muthukrishnan S. (2016). Biosynthesis, turnover, and functions of chitin in insects. Annu. Rev. Entomol..

[14] Anitha A., Sowmya S., Kumar P.T.S., Deepthi S., Chennazhi K.P., Ehrlich H., Tsurkan M., Jayakumar R. (2014). Chitin and chitosan in selected biomedical applications. Prog. Polym. Sci..

[15] Jayakumar R., Chennazhi K.P., Srinivasan S. (2011). Chitin scaffolds in tissue engineering. Int. J. Mol. Sci..

[16] Zhang X., Rolandi M. (2017). Engineering strategies for chitin nanofibers. J. Mater. Chem. B.

[17] Di Mario F., Rapana P., Tomati U., Galli E. (2008). Chitin and chitosan from basidiomycetes. Int. J. Biol. Macromol..

[18] Hassainia A., Satha H., Boufi S. (2018). Chitin from *Agaricus bisporus*: Extraction and characterization. Int. J. Biol. Macromol..

[19] Nawawi W.M.F.W., Lee K.Y., Kontturi E., Murphy R.J., Bismarck A. (2019). Chitin nanopaper from mushroom extract: Natural composite of nanofibers and glucan from a single biobased source. ACS Sustain. Chem. Eng..

[20] van Leeuwen J., Norton G.A., Ndlela S.S., Rudnick D. (2016). Processes for Isolating Chitin and Chitosan from Fungal Biomass. U.S. Patents.

[21] Wu T., Zivanovic S., Draughon F.A., Conway W.S., Sams C.E. (2005). Physicochemical properties and bioactivity of fungal chitin and chitosan. J. Agric. Food Chem..

[22] Zeng J.B., He Y.S., Li S.L., Wang Y.Z. (2012). Chitin whiskers: An overview. Biomacromolecules.

[23] Chow K.S., Khor E. (2000). Novel fabrication of open-pore chitin matrixes. Biomacromolecules.

[24] Shaala L.A., Asfour H.Z., Youssef D.T.A., Zółtowska-Aksamitowska S., Wysokowski M., Tsurkan M., Galli R., Meissner H., Petrenko I., Tabachnick K. (2019). New source of 3D chitin scaffolds: The Red Sea Demosponge *Pseudoceratina arabica* (Pseudoceratinidae, Verongiida). Mar. Drugs.

[25] Wysokowski M., Petrenko I., Stelling A.L., Stawski D., Jesionowski T., Ehrlich H. (2015). Poriferan chitin as a versatile template for extreme biomimetics. Polymers (Basel).

[26] Zółtowska-Aksamitowska S., Shaala L.A., Youssef D.T.A., Elhady S.S., Tsurkan M.V., Petrenko I., Wysokowski M., Tabachnick K., Meissner H., Ivanenko V.N. (2018). First report on chitin in a non-verongiid marine demosponge: The *Mycale euplectellioides* case. Mar. Drugs.

[27] Żółtowska-Aksamitowska S., Tsurkan M.V., Lim S.C., Meissner H., Tabachnick K., Shaala L.A., Youssef D.T.A., Ivanenko V.N., Petrenko I., Wysokowski M. (2018). The demosponge *Pseudoceratina purpurea* as a new source of fibrous chitin. Int. J. Biol. Macromol..

[28] Schleuter D., Günther A., Paasch S., Ehrlich H., Kljajić Z., Hanke T., Bernhard G., Brunner E. (2013). Chitin-based renewable materials from marine sponges for uranium adsorption. Carbohydr. Polym..

[29] Stepniak I., Galinski M., Nowacki K., Wysokowski M., Jakubowska P., Bazhenov V.V., Leisegang T., Ehrlich H., Jesionowski T. (2016). A novel chitosan/sponge chitin origin material as a membrane for supercapacitors-preparation and characterization. RSC Adv..

[30] Ehrlich H., Ruys A.J. (2013). Biomimetic potential of chitin-based composite biomaterials of poriferan origin. Biomimetic Biomaterials: Structure and Applications.

[31] Petrenko I., Bazhenov V.V., Galli R., Wysokowski M., Fromont J., Schupp P.J., Stelling A.L., Niederschlag E., Stöker H., Kutsova V.Z. (2017). Chitin of poriferan origin and the bioelectrometallurgy of copper/copper oxide. Int. J. Biol. Macromol..

[32] Wysokowski M., Materna K., Walter J., Petrenko I., Stelling A.L., Bazhenov V.V., Klapiszewski Ł., Szatkowski T., Lewandowska O., Stawski D. (2015). Solvothermal synthesis of hydrophobic chitin-polyhedral oligomeric silsesquioxane (POSS) nanocomposites. Int. J. Biol. Macromol..

[33] Wysokowski M., Motylenko M., Beyer J., Makarova A., Stöcker H., Walter J., Galli R., Kaiser S., Vyalikh D., Bazhenov V.V. (2015). Extreme biomimetic approach for developing novel chitin-GeO_2_ nanocomposites with photoluminescent properties. Nano Res..

[34] Wysokowski M., Szalaty T.J., Jesionowski T., Motylenko M., Rafaja D., Koltsov I., Stöcker H., Bazhenov V.V., Ehrlich H., Stelling A.L. (2017). Extreme biomimetic approach for synthesis of nanocrystalline chitin-(Ti,Zr)O_2_ multiphase composites. Mater. Chem. Phys..

[35] Steck E., Burkhardt M., Ehrlich H., Richter W. (2010). Discrimination between cells of murine and human origin in xenotransplants by species specific genomic in situ hybridization. Xenotransplantation.

[36] Mutsenko V.V., Gryshkov O., Lauterboeck L., Rogulska O., Tarusin D.N., Bazhenov V.V., Schütz K., Brüggemeier S., Gossla E., Akkineni A.R. (2017). Novel chitin scaffolds derived from marine sponge *Ianthella basta* for tissue engineering approaches based on human mesenchymal stromal cells: Biocompatibility and cryopreservation. Int. J. Biol. Macromol..

[37] Mutsenko V.V., Bazhenov V.V., Rogulska O., Tarusin D.N., Schütz K., Brüggemeier S., Gossla E., Akkineni A.R., Meißner H., Lode A. (2017). 3D chitinous scaffolds derived from cultivated marine demosponge *Aplysina aerophoba* for tissue engineering approaches based on human mesenchymal stromal cells. Int. J. Biol. Macromol..

[38] Ehrlich H., La Barre S., Bates S.S. (2018). Chitin of Poriferan Origin as a Unique Biological Material. Blue Biotechnology: Production and Use of Marine Molecules.

[39] Ehrlich H., Maldonado M., Parker A.R., Kulchin Y.N., Schilling J., Köhler B., Skrzypczak U., Simon P., Reiswig H.M., Tsurkan M.V. (2016). Supercontinuum generation in naturally occurring glass sponges spicules. Adv. Opt. Mater..

[40] Vette R.S., Rust M.K. (2010). Periodicity of molting and resumption of post-molt feeding in the brown recluse spider *Loxosceles reclusa* (Araneae: Sicariidae). J. Kansas Entomol. Soc..

[41] Machałowski T., Wysokowski M., Żółtowska-Aksamitowska S., Bechmann N., Binnewerg B., Schubert M., Guan K., Bornstein S.R., Czaczyk K., Pokrovsky O. (2019). Spider Chitin. The biomimetic potential and applications of *Caribena versicolor* tubular chitin. Carbohydr. Polym..

[42] Bagaturov M.F., Jammu A. (2005). Some notes on breeding *Avicularia versicolor* with comments on the hobby in Russia. J. Br. Tarantula Soc..

[43] Global Tarantula Market. https://www.globaltarantulamarket.com/.

[44] German Arachnologic Society. https://www.dearge.de/english.php.

[45] British Tarantula Society. https://www.thebts.co.uk/.

[46] Platnick N.I. World Spider Catalog (2019). http://wsc.nmbe.ch.

[47] Collatz K.G., Mommsen T. (1975). Physiological conditions and variations of body constituents during the moulting cycle of the spider *Tegenaria atrica* C. L. Koch (agelenidae). Comp. Biochem. Physiol. Part A.

[48] Minch E.W. (1977). The molting sequence in *Aphonopelma chalcodes* (Araneae: Theraphosidae). J. Arachnol..

[49] Nyffeler M., Birkhofer K. (2017). An estimated 400–800 million tons of prey are annually killed by the global spider community. Sci. Nat..

[50] Cloudsley-Thompson J.L. (1950). Epicuticle of arthropods. Nature.

[51] Rudall K.M. (1963). The chitin/protein complexes of Insect cuticles. Adv. Insect Physiol..

[52] Sewell M.T. (1951). Pore canals in a spider cuticle. Nature.

[53] Sewell M.T. (1955). The histology and histochemistry of the cuticle of a spider, *Tegenaria domestica*. Ann. Entomol. Soc. Am..

[54] Nentwig W. (1987). Ecophysiology of Spiders.

[55] Davies G., Knight D.P., Vollrath F. (2013). Chitin in the silk gland ducts of the spider *Nephilia edulis* and silkworm *Bombyx mori*. PLoS ONE.

[56] Hsiung B.-K., Blackledge T.A., Shawkey M.D. (2015). Spiders do have melanin after all. J. Exp. Biol..

[57] Tarangini K., Mishra S. (2014). Production of melanin by soil microbial isolate on fruit waste extract: Two step optimization of key parameters. Biotechnol. Rep..

[58] Casadevall A. (2018). Melanin triggers antifungal defences. Nature.

[59] Łopusiewicz Ł. (2018). The isolation, purification and analysis of the melanin pigment extracted from *Armillaria mellea* rhizomorphs. World Sci. News.

[60] Roy S., Rhim J. (2019). Preparation of carrageenan-based functional nanocomposite films incorporated with melanin nanoparticles. Colloids Surf. B Biointerfaces.

[61] Zhao H., Zeng Z., Liu L., Chen J., Zhou H., Huang L., Huang J., Xu H., Xu Y., Chen Z. (2018). Polydopamine nanoparticles for treatment of acute inflammation-induced injury. Nanoscale.

[62] Solano F. (2017). Melanin and melanin-related polymers as materials with biomedical and biotechnological applications — cuttlefish ink and mussel foot proteins as inspired biomolecules. Int. J. Mol. Sci..

[63] Mbonyiryivuze A., Mwakikunga B., Dhlamini S.M., Maaza M. (2015). Fourier transform infrared spectroscopy for Sepia melanin. Phys. Mater. Chem..

[64] Politi Y., Priewasser M., Pippel E., Zaslansky P., Hartmann J., Siegel S., Li C., Barth F.G., Fratzl P. (2012). A Spider’s Fang: How to design an injection needle using chitin-based composite material. Adv. Funct. Mater..

[65] Politi Y., Pippel E., Licuco-Massouh A.C.J., Bertinetti L., Blumtritt H., Barth F.G., Fratzl P. (2017). Nano-channels in the spider fang for the transport of Zn ions to cross-link His-rich proteins pre-deposited in the cuticle matrix. Arthropod Struct. Dev..

[66] Fukushima C.S., Bertani R. (2017). Taxonomic revision and cladistic analysis of *Avicularia* Lamarck, 1818 (Araneae, Theraphosidae, Aviculariinae) with description of three new aviculariine genera. Zookeys.

[67] Bertani R., Boston T., Evenou Y., Huadanucci J.P.L. (2003). Urticating hairs of *Avicularia versicolor* (Walckenaer, 1837) (Araneae, Theraphosidae). Br. Arachnol. Soc..

[68] Browning H.C. (1942). The integument and moult cycle of *Tegenaria atrica* (Araneae). Proc. R. Soc. B.

[69] Nemenz H. (1955). Über den Bau der Kutikula und dessen Einfluß auf die Wasserabgabe bei Spinnen. Osterr Akad Wiss Math-Nat KI Abt I.

[70] Barth F.G. (1969). Die Feinstruktur des Spinneninteguments, I. Die Cuticula des Laufbeins adulter hiiutungsferner Tiere (*Cupiennius salei* Keys). Zeitschrift für Zellforschung und Mikroskopische Anatomie.

[71] Barth F.G. (1970). Die Feinstruktur des Spinneninteguments - II. Die raumliche Anordnung der Mikrofasern in der lamellierten Cuticula und ihre Beziehung zur Gestalt der Porenkanale (Cupiennius salei Keys., adult, hautungsfern, Tarsus). Zeitschrift für Zellforschung und Mikroskopische Anatomie.

[72] Barth F.G. (1973). Microfiber reinforcement of an arthropod cuticle. Cell Tissue Res..

[73] Hadley N.F. (1978). Cuticular permeability and lipid composition of the black widow spider, *Latrodectus hesperus*. Proc. R. Soc. B.

[74] Hadley N.F. (1981). Fine structure of the cuticle of the vlack window spider with reference to surface lipids. Tissue Cell.

[75] Krishnakumaran A. (1960). The early post-moult cuticle in Buthus. Q. J. Microsc. Sci..

[76] Krishnakumaran A. (1961). A comparative study of the Atachnid cuticle. II Chemical nature. Zeitschrift fur vergleiehende Physiol..

[77] Krishnakumaran A. (1962). A comparative study of the cuticle in Arachnida. I. Structure and staining properties. Zool. Jahrb. Anat..

[78] Klinger C., Żółtowska-Aksamitowska S., Wysokowski M., Tsurkan M.V., Galli R., Petrenko I., Machałowski T., Ereskovsky A., Martinović R., Muzychka L. (2019). Express hethod for isolation of ready-to-use 3D chitin scaffolds from *Aplysina archeri* (Aplysineidae: Verongiida) Demosponge. Mar. Drugs.

[79] Kintsu H., Okumura T., Negishi L., Ifuku S., Kogure T., Sakuda S., Suzuki M., Endo K., Kogure T., Nagasawa H. (2018). Chitin degraded by chitinolytic enzymes induces crystal defects of calcites. Biomineralization.

[80] Yeng Y.A.L., Kadir S.M.A., Ghazali H.M., Rahman R.N.Z.R., Saari N. (2013). A comparative study of extraction techniques for maximum recovery of glutamate decarboxylase (GAD) from *Aspergillus oryzae* NSK. BMC Res. Notes.

[81] Ehrlich H., Wysokowski M., Zółtowska-Aksamitowska S., Petrenko I., Jesionowski T. (2018). Collagens of poriferan origin. Mar. Drugs.

[82] Fromont J., Żółtowska-Aksamitowska S., Galli R., Meissner H., Erpenbeck D., Vacelet J., Diaz C., Tsurkan M.V., Petrenko I., Youssef D.T.A. (2019). New family and genus of a Dendrilla-like sponge with characters of Verongiida. Part II. Discovery of chitin in the skeleton of *Ernstilla lacunosa*. Zool. Anz..

[83] Gómez-Ordóñez E., Rupérez P. (2011). FTIR-ATR spectroscopy as a tool for polysaccharide identification in edible brown and red seaweeds. Food Hydrocoll..

[84] Shang S., Zhu L., Fan J. (2013). Intermolecular interactions between natural polysaccharides and silk fibroin protein. Carbohydr. Polym..

[85] Machovic V., Lapcak L., Havelcova M., Borecka L., Novotna M., Novotna M., Javurkova I., Langrova I., Hajkova S., Brozova A. (2017). Analysis of European honeybee (*Apis mellifera*) wings using ATR-FTIR and Raman spectroscopy: A pilot study. Sci. Agric. Bohem..

[86] Focher B., Naggi A., Torri G., Cossani A., Terbojevich M. (1992). Structural differences between chitin polymorphs and their precipitates from solutions – evidence from CP-MAS ^13^C NMR, FT-IR and FT-Raman spectroscopy. Carbohydr. Polym..

[87] Minke R., Blackwell J. (1978). The structure of α-chitin. J. Mol. Biol..

[88] Nishiyama Y., Noishiki Y., Wada M. (2011). X-ray structure of anhydrous β-chitin at 1A resolution. Macromolecules.

[89] Jang M., Kong B., Jeong Y., Lee C.H., Nah J. (2004). Physicochemical characterization of α-chitin, β-chitin, and γ-chitin separated from natural resources. J. Polym. Sci. Part A Polym. Chem..

[90] Sikorski P., Hori R., Wada M. (2009). Revisit of α-chitin crystal structure using high resolution X-ray diffraction data. Biomacromolecules.

[91] Ingersoll H.G. (1946). Fine structure of viscose rayon. J. Appl. Phys..

[92] Segal L., Creely J.J., Martin J., Conrad C.M. (1958). An empirical method for estimating the degree of crystallinity of native cellulose using the X-ray diffractometer. Text. Res. J..

[93] Struszczyk H. (1987). Microcrystalline chitosan. I. Preparation and properties of microcrystalline chitosan. J. Appl. Polym. Sci..

[94] Focher B., Beltrame P.L., Naggi A., Torri G. (1990). Alkaline N-deacetylation of chitin enhanced by flash treatments. Reaction kinetics and structure modifications. Carbohydr. Polym..

[95] Hong A.S., Yang Q., Yuan Y., Chen L., Song Y., Lian H. (2019). Sustainable co-solvent induced one step extraction of low molecular weight chitin with high purity from raw lobster shell. Carbohydr. Polym..

[96] Andrade C.T., Silva K.M.P., Tavares M.I., Simao R.A., Achete C., Perez C.A. (2001). Comparative study on structural features of α chitin from *Xiphopenaeus kroyeri* and its precipitated product from phosporic acid solution. J. Appl. Polym. Sci..

[97] Neinhuis C., Nickerl J., Tsurkan M., Werner C., Werner C. (2014). The multi-layered protective cuticle of Collembola: A chemical analysis. J. R. Soc. Interface.

[98] Ehrlich H., Rigby J.K., Botting J.P., Tsurkan M.V., Werner C., Schwille P., Petrášek Z., Pisera A., Simon P., Sivkov V.N. (2013). Discovery of 505-million-year old chitin in the basal demosponge *Vauxia gracilenta*. Sci. Rep..

[99] Domszya J.G., Roberts G.A.F. (1985). Evaluation of infrared spectroscopic techniques for analysing chitosan. Macromol. Chem. Phys..

[100] Knidri H.E.L., Khalfaouy R.E.L., Laajeb A., Addaou A., Lahsini A. (2016). Eco-friendly extraction and characterization of chitin and chitosan from the shrimp shell waste via microwave irradiation. Process. Saf. Environ. Prot..

[101] Małecki P.H., Raczynska J., Vorgias C.E., Rypniewski W. (2013). Structure of a complete four-domain chitinase from *Moritella marina*, a marine psychrophilic bacterium. Acta Crystallogr. Sect. D Biol. Crystallogr..

[102] Papadimitriou K., Baharidis P.K., Georgoulis A., Engel M., Louka M., Karamolegkou G., Tsoka A., Blom J., Pot B., Malecki P. (2016). Analysis of the complete genome sequence of the archaeon *Pyrococcus chitonophagus* DSM 10152 (formerly *Thermococcus chitonophagus*). Extremophiles.

