# Supplementary material for: Spider Chitin: An Ultrafast Microwave-Assisted Method for Chitin Isolation from Caribena versicolor Spider Molt Cuticle
Source: Molecules. 2019 Oct 16;24(20):3736. doi: 10.3390/molecules24203736 (PMC6833065; doi:10.3390/molecules24203736)
Supplement: Supplementary file 1 [file molecules-24-03736-s001.pdf]

molecules

Supporting information to:

Spider Chitin: An ultrafast microwave-assisted method for chitin isolation from *Caribena versicolor* spider molt cuticle

Tomasz Machałowski [1,2], Marcin Wysokowski [1,2], Mikhail V. Tsurkan [3], Roberta Galli [4], Christian Schimpf [5], David Rafaja [5], Erica Brendler [6], Christine Viehweger [6], Sonia Żółtowska-Aksamitowska [1,2], Iaroslav Petrenko [2], Katarzyna Czaczyk [7], Michael Kraft [8], Martin Bertau [8], Nicole Bechmann [9], Kaomei Guan [10], Stefan R. Bornstein [11,12], Alona Voronkina [13], Andriy Fursov [2], Magdalena Bejger [14], Katarzyna Biniek-Antosiak [14], Wojciech Rypniewski [14], Marek Figlerowicz [14], Oleg Pokrovsky [15, 16], Teofil Jesionowski [1,*], Hermann Ehrlich [2,*]

[1] Institute of Chemical Technology and Engineering, Faculty of Chemical Technology, Poznan University of Technology, Poznan 60965, Poland
[2] Institute of Electronics and Sensor Materials, TU Bergakademie Freiberg, Freiberg 09599, Germany
[3] Leibniz Institute of Polymer Research Dresden, Dresden 01069, Germany
[4] Clinical Sensoring and Monitoring, Department of Anesthesiology and Intensive Care Medicine, Faculty of Medicine, TU Dresden, Dresden 01307, Germany
[5] Institute of Materials Science, TU Bergakademie Freiberg, Freiberg 09599, Germany
[6] Institute of Analytical Chemistry, TU Bergakademie Freiberg, Freiberg 09599, Germany
[7] Department of Biotechnology and Food Microbiology, Poznan University of Life Sciences, Poznan 60637, Poland
[8] Institute of Chemical Technology, TU Bergakademie Freiberg, Freiberg 09599, Germany
[9] Institute of Clinical Chemistry and Laboratory Medicine, University Hospital Carl Gustav Carus, TU Dresden, Dresden 01307, Germany
[10] Institute of Pharmacology and Toxicology, TU Dresden, Dresden 01307, Germany
[11] Center for Regenerative Therapies Dresden, TU Dresden, Dresden 01307, Germany
[12] Department of Medicine III, University Hospital Carl Gustav Carus Dresden, TU Dresden, Dresden 01307, Germany
[13] Department of Pharmacy, National Pirogov Memorial Medical University, Vinnytsia 21018, Ukraine
[14] Institute of Bioorganic Chemistry, Polish Academy of Sciences, Poznan 61704, Poland
[15] Geoscience and Environment Toulouse, UMR 5563 CNRS, Toulouse 31400, France
[16] BIO-GEO-CLIM Laboratory, Tomsk State University, Tomsk, Russia
* Correspondence: Teofil.Jesionowski@put.poznan.pl; Tel.: +48-517-383-325, Hermann.Ehrlich@esm.tu-freiberg.de; Tel.: +49-177-2582-179

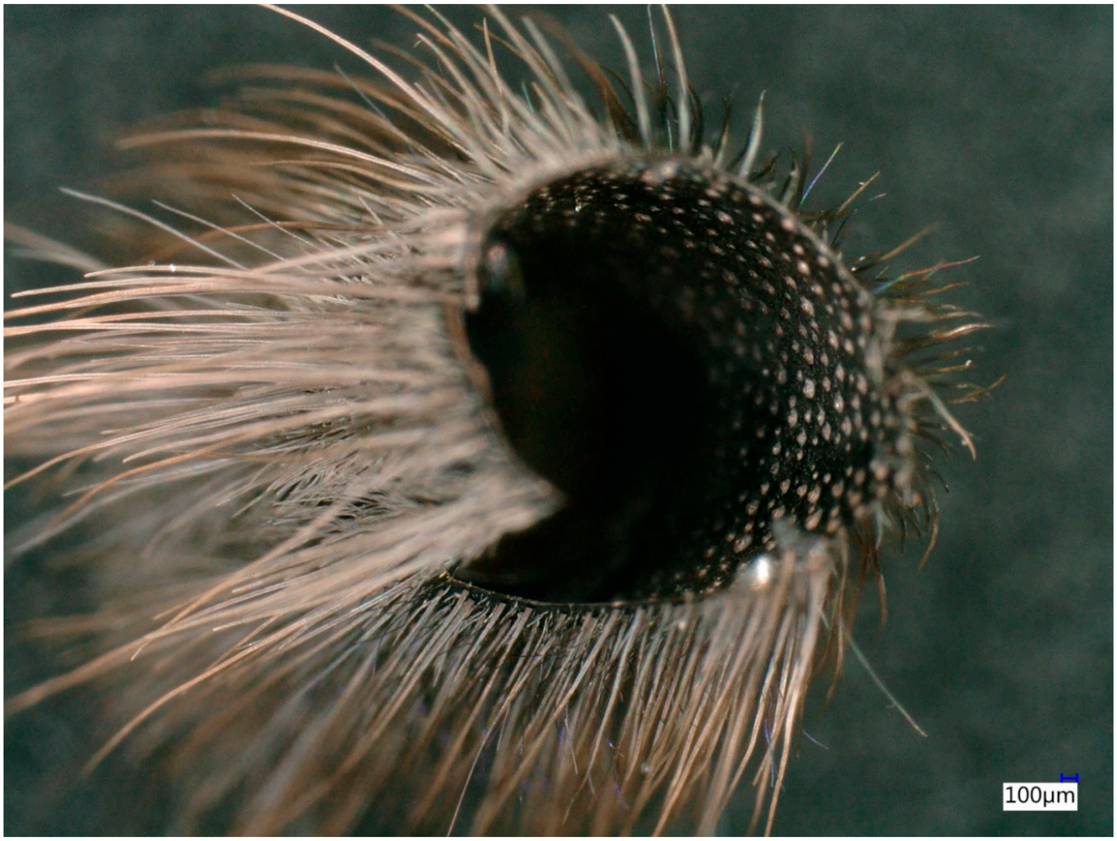

Figure S1. Molt cuticle from the walking leg of *C. versicolor* spider represents up to 3 cm-long "brushy tube".

The samples were observed and photographed (not shown) under the microscope. No visual changes were noticed. Then samples were taken of the solutions and analyzed by gas chromatography coupled to mass spectrometry (GC-MS). No NAG products of chitinase activity were detected in comparison to control experiments (i.e. incubations carried out in the absence of chitinases). The activity of the used chitinases was previously confirmed but additional tests were performed immediately after the above negative results, on two of the enzymes (Chi60 and its deletion mutant) using a freshly purchased Chitinase Assay Kit (Sigma). The control activity test showed the enzymes to be active against the standard assay substrate 4-Nitrophenyl N,N′-diacetyl-β-D-chitobioside (pNP-NAG2). This confirms that isolated cuticle is resistant to four chitinases: Chi60 from *M.marina* and its deletion mutant, missing two Ig-like domains [1] and two chitinases, Chiton_1119 and Chiton_1716, from *P.chitonophagus*.

Only Yatalase solution ensures proper enzymatic digestion of isolated chitin cuticle. Yatalase is a complex enzyme, consisting mainly of chitinase, chitobiase and β-1,3-glucanase. One unit of this enzyme released one µmol of N-acetylglucosamine from 0.5% chitin solution and 1 µmol of p-nitrophenol from p-nitrophenyl-N-acetyl-β-D-glucosaminide solution in 1 min at 37 °C.

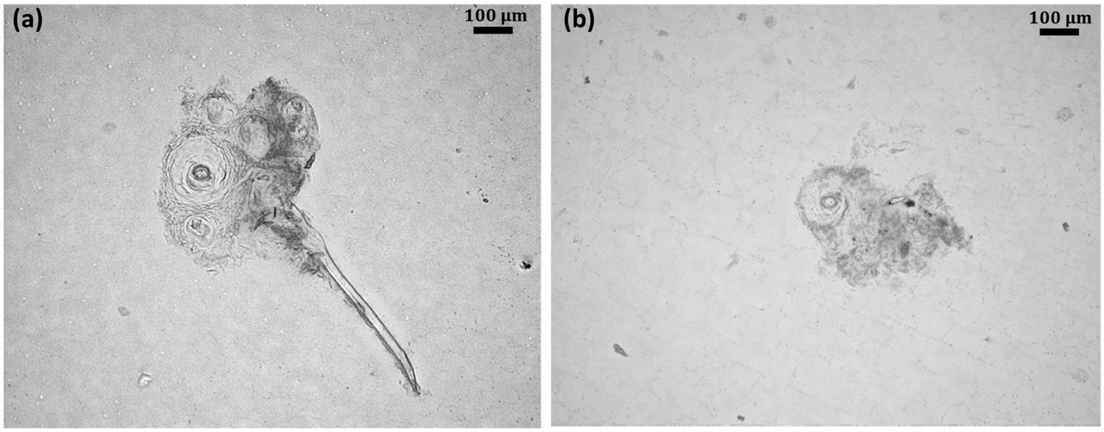

Figure S2. Chitinase test of chitin obtained from *C. versicolor* using a microwave-assisted method. (a) A piece of chitinous cuticle with setae is partially dissolved (b) after immersion for 6 hours in the Yatalase solution at 37 °C.

The results of ATR-FTIR analysis of the molt cuticle at various stages of isolation are presented in Figure S3. The spectrum obtained for the final transparent, insoluble scaffold shows a characteristic split of the amide I band (mainly associated with C=O stretching vibrations) with peaks at 1654 and 1619 cm^{-1} (gray line). Splitting of the amide I band results from the presence of stretching vibrations from intermolecular (C=O···HN) and intramolecular (C=O···HO(C6); C=O···HN) hydrogen bonds and is characteristic and distinctive for α-chitin [2,3]. The presence of such non-equivalent populations of C=O groups is also shown by Raman spectroscopy (Figure 6) and corresponds with previously published experimental data for α-chitin [2–4]. Additional features such as the presence of amide II (νN-H and νC-N), amide III (νC–N and δN–H), and characteristic intense bands at 951 cm^{-1} (ωCH_x) and 895 cm^{-1} (β-glycosidic bonds) are observed in the spectrum of the isolated scaffold and also correspond with data reported for α-chitin [2–5]. According to calculations (equation 3 and equation 4, main manuscript) the degree of acetylation (DA%) and degree of deacetylation (DD%) were determined at DA=99 % and DD=1 %. This confirms that the proposed treatment, due to its very short duration, does not have a negative impact on the degree of acetylation.

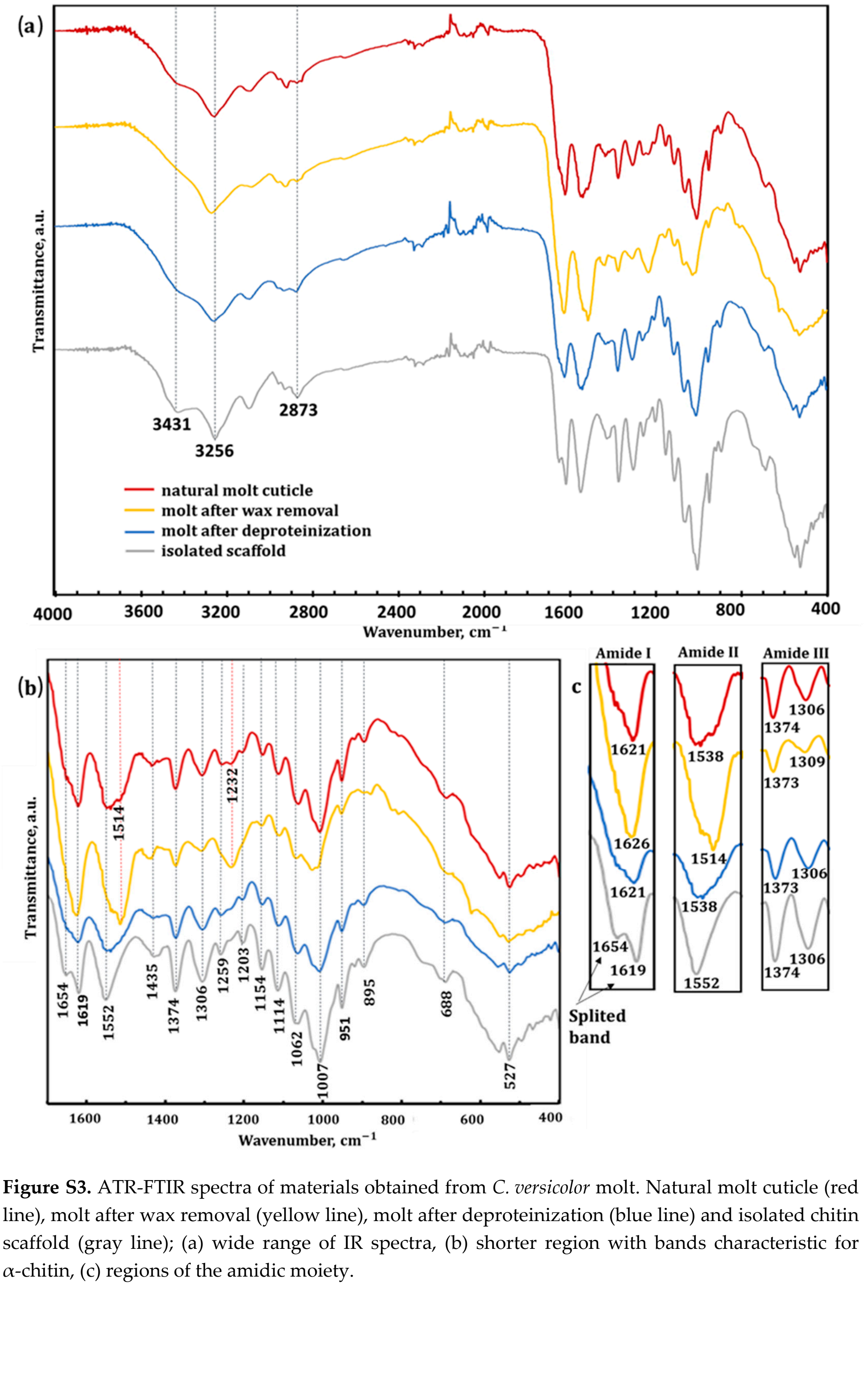

Figure S3. ATR-FTIR spectra of materials obtained from *C. versicolor* molt. Natural molt cuticle (red line), molt after wax removal (yellow line), molt after deproteinization (blue line) and isolated chitin scaffold (gray line); (a) wide range of IR spectra, (b) shorter region with bands characteristic for α-chitin, (c) regions of the amidic moiety.

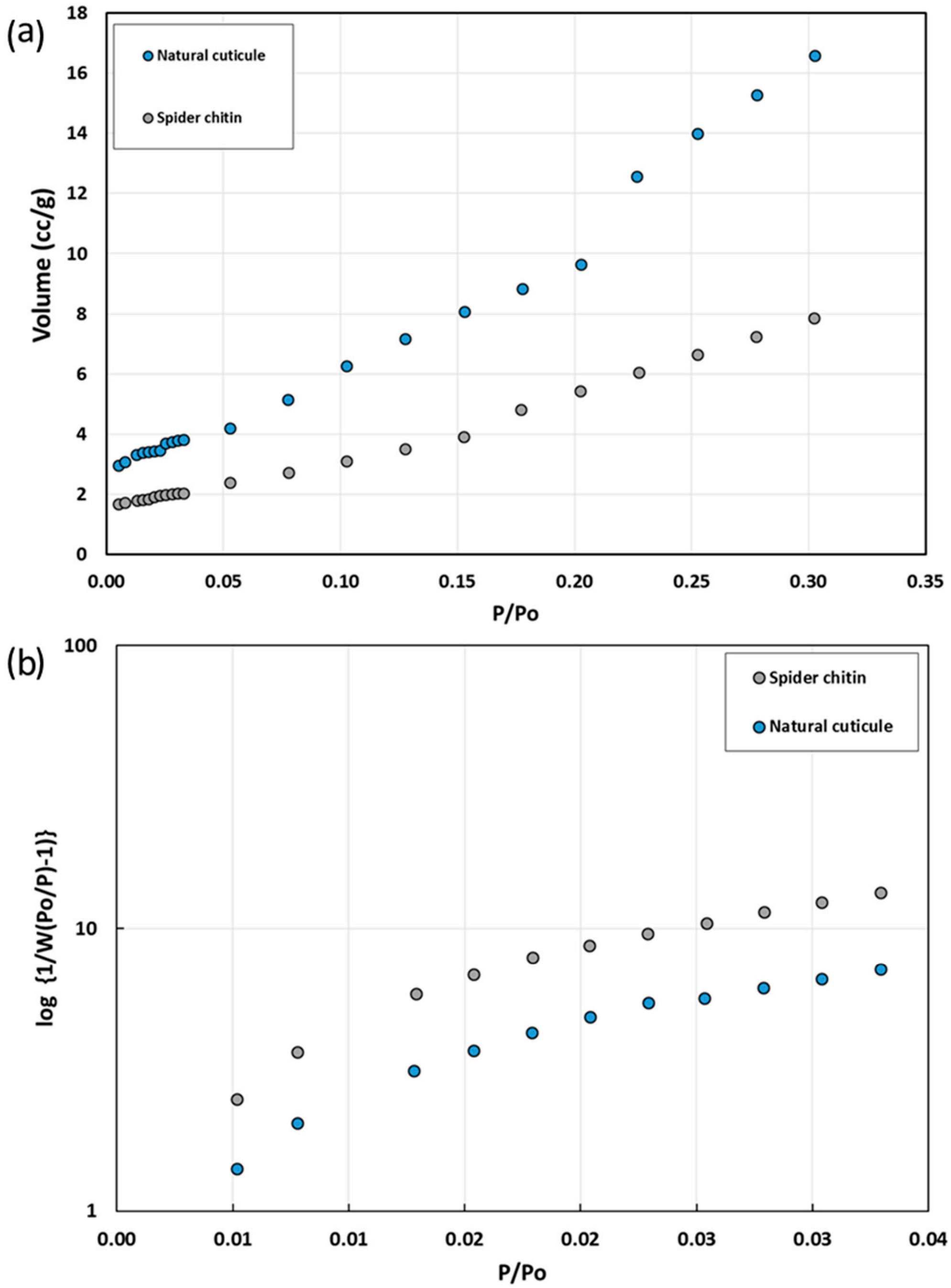

Figure S4. (a) N_2 adsorption isotherm for natural cuticle and isolated chitin; (b) P/Po versus log A/(W((Po/P)-1)) for natural cuticle and isolated chitin.

Identification of pigment

The quantity of pigment powder obtained after the deproteinization step (see 3.2.3.) was determined at 8 % of the mass of the molt. UV-VIS spectra of the *C. versicolor* cuticular pigment were compared with standard melanin from *S. officinalis* (Figure S5). Decreases in UV absorption characteristic for melanins are observed for both pigments. The maxima of absorption fall in the UV region, and it then rapidly decreases in the VIS region (Figure S5). This phenomenon is characteristic of melanin [6]. In contrast to *Sepia* melanin, the maximum absorption of the spider pigment is observed around λ = 315 nm, which fits extremely well to 5,6-dihydroxyindole-2-carboxylic acid (DHICA)

eumelanin-like pigment [7–9]. The very low absorption in the visible region may suggest the presence of a reduced form of quinonoid chromophores [7].

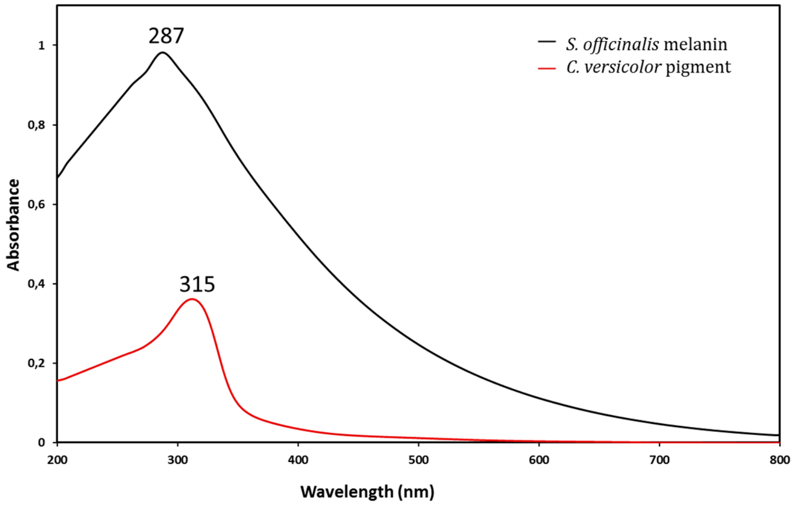

Figure S5. Absorption spectrum of melanin-like pigment isolated from *C. versicolor* (Theraphosidae) spider and standard *S. officinalis* melanin.

Additionally, we performed identification of the melanin-based pigment in the naturally occurring molt of *C. versicolor* using infrared spectroscopy. FTIR spectra of the spider pigment and melanin isolated from *S. officinalis* are shown in Figure S6. Peaks recorded in the 3600–2950 cm^{-1} range are attributed to stretching vibrations (O–H and N–H) of the amine, amide, or carboxylic acid, phenolic and aromatic amino functional groups present in the indolic and pyrrolic systems [10]. The bands at 2930 cm^{-1} and 2872 cm^{-1} are associated with stretching vibrations of C–H from CH and CH_2 [11]. The band at 1633 cm^{-1} can be attributed to stretching vibrations of C = O and/or of COO^- groups [10,11]. The N-H bending vibration peak at 1543 cm^{-1} indicates that the pigment has the typical indole structure of melanin [10]. The band at 1514 cm^{-1} is associated with the C–C stretching vibrations in aromatic rings. On the spectrum of the isolated pigment, the clearly visible peak at 1386 cm^{-1} related to C–N stretching is characteristic of the aromatic fundamental units of the pyrrole or indole ring [12]. Bending vibrations of C–CH_3 were recorded at 1109 cm^{-1}. The bands at 1236 cm^{-1} and 1040 cm^{-1} are associated with vibrations of phenolic C–O [13]. The spectrum recorded for the spider pigment is similar to that of melanin isolated from *S. officinalis* and corresponds with experimental data reported in the literature [10,11,13,14]. On the basis of the above results (Figure S6) it is suggested that the obtained pigment belongs to the group of melanins.

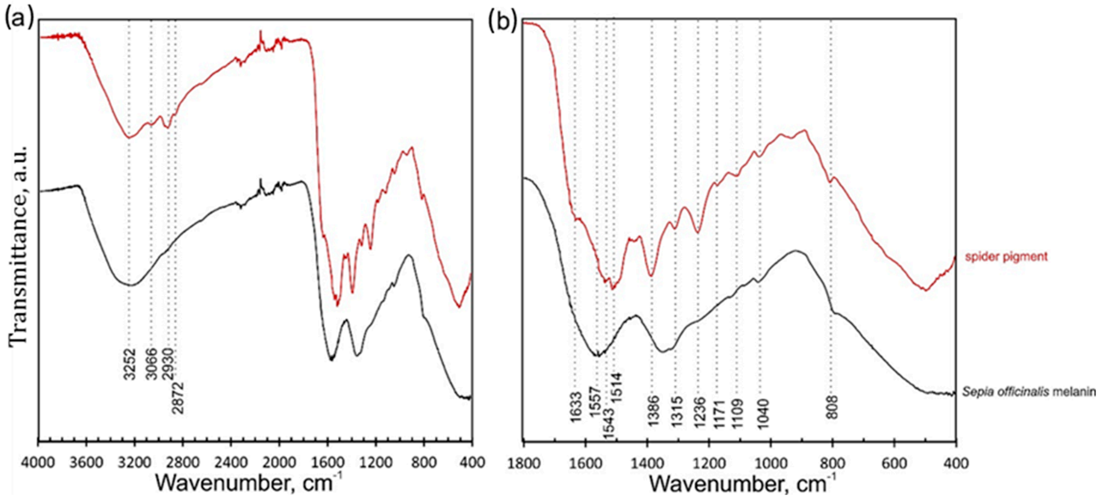

Figure S6. ATR-FTIR spectra of pigment powder obtained from *C. versicolor* (Theraphosidae) molt (red line), and melanin powder of *S. officinalis* (black line). (a) Wide range of IR spectra, (b) shorter region with bands characteristic for melanin-like materials .

References

1. Małecki, P.H.; Raczynska, J.; Vorgias, C.E.; Rypniewski, W. Structure of a complete four-domain chitinase from *Moritella marina*, a marine psychrophilic bacterium. *Acta Crystallogr. Sect. D Biol. Crystallogr.* **2013**, *69*, 821–829.

2. Focher, B.; Naggi, A.; Torri, G.; Cossani, A.; Terbojevich, M. Structural differences between chitin polymorphs and their precipitates from solutions – evidence from CP-MAS ^{13}C NMR, FT-IR and FT-Raman spectroscopy. *Carbohydr. Polym.* **1992**, *17*, 97–102.

3. Kumirska, J.; Czerwicka, M.; Kaczyński, Z.; Bychowska, A.; Brzozowski, K.; Thöming, J.; Stepnowski, P. Application of spectroscopic methods for structural analysis of chitin and chitosan. *Mar. Drugs* **2010**, *8*, 1567–1636.

4. Kaya, M.; Mujtaba, M.; Ehrlich, H.; Salaberria, A.M.; Baran, T.; Amemiya, C.T.; Galli, R.; Akyuz, L.; Sargin, I.; Labidi, J. On chemistry of γ-chitin. *Carbohydr. Polym.* **2017**, *176*, 177–186.

5. Huang, W.; Zhao, D.; Guo, N.; Xue, C.; Mao, X. Green and facile production of chitin from crustacean shells using a natural deep eutectic solvent. *J. Agric. Environ. Chem.* **2018**, *66*, 11897–11901.

6. Tarangini, K.; Mishra, S. Production of melanin by soil microbial isolate on fruit waste extract : two step optimization of key parameters. *Biotechnol. Reports* **2014**, *4*, 139–146.

7. Micillo, R.; Panzella, L.; Koike, K.; Monfrecola, G.; Napolitano, A.; Ischia, M. " Fifty shades " of black and red or how carboxyl groups fine tune eumelanin and pheomelanin properties. *Int. J. Mol. Sci.* **2016**, *17*, 746.

8. Olaizola, C.; Abramowski, Z.A.; Jovel Ayala, E.M. Photoprotective effect of fungal melanins against UVB in human skin cells. *Microbiol. Apl. Int.* **2013**, *25*, 3–12.

9. Pezzella, A.; Iadonisi, A.; Valerio, S.; Panzella, L.; Napolitano, A.; Adinolfi, M.; Ischia, M. Disentangling eumelanin " black chromophore": visible absorption changes as signatures of oxidation state- and aggregation-dependent dynamic interactions in a model water-soluble 5,6-dihydroxyindole polymer. *J. od Am. Chem. Soc.* **2009**, *131*, 15270–15275.

10. El-Naggar, N.E.-A.; El-Ewasy, S.M. Bioproduction, characterization, anticancer and antioxidant activities of extracellular melanin pigment produced by newly isolated microbial cell factories *Streptomyces glaucescens* NEAE-H. *Sci. Rep.* **2017**, *7*, 42129.

11. Liu, Y.; Zheng, W.; Ibrahim, S.A.; Yang, H.; Huang, W. Chemical properties of vacuum-fried *Pleurotus eryngii* during storage and characterization of brown pigment. *Int. J. Food Prop.* **2018**, *20*, S2349–S2358.

12. Bridelli, M.G.; Crippa, P.R. Infrared and water sorption studies of the hydration structure and mechanism in natural and synthetic melanin. *J. Phys. Chem. B* **2010**, *114*, 9381–9390.

13. Drewnowska, J.M.; Zambrzycka, M.; Kalska-Szostko, B.; Fiedoruk, K.; Swiecicka, I. Melanin-like pigment synthesis by soil *Bacillus weihenstephanensis* isolates from northeastern Poland. *PLoS One* **2015**, *10*, e0125428.

14. Pinto, L.; Fernando, L.; Granja, Z.; Almeida, M.A. De; Alviano, D.S.; Helena, M.; Ejzemberg, R.; Rozental, S.; Alviano, C.S. Melanin particles isolated from the fungus *Fonsecaea pedrosoi* activates the human complement system. *Mem. Inst. Oswaldo Cruz* **2018**, *113*, e180120.

© 2019 by the authors. Submitted for possible open access publication under the terms and conditions of the Creative Commons Attribution (CC BY) license (http://creativecommons.org/licenses/by/4.0/).
